# Both mitochondrial DNA and mitonuclear gene mutations cause hearing loss through cochlear dysfunction

**DOI:** 10.1093/brain/aww051

**Published:** 2016-03-25

**Authors:** Peter J. Kullar, Jenna Quail, Phillip Lindsey, Janet A. Wilson, Rita Horvath, Patrick Yu-Wai-Man, Grainne S. Gorman, Robert W. Taylor, Yi Ng, Robert McFarland, Brian C. J. Moore, Patrick F. Chinnery

**Affiliations:** ^1^ Department of Clinical Neuroscience, School of Clinical Medicine, University of Cambridge, Cambridge CB2 0QQ, UK; ^2^ Medical Research Council Mitochondrial Biology Unit, Cambridge Biomedical Campus, Cambridge, CB2 0QQ, UK; ^3^ Freeman Hospital, Freeman Road, Newcastle-upon-Tyne, NE7 7DN, UK; ^4^ Wellcome Trust Centre for Mitochondrial Research, Institute of Genetic Medicine, Newcastle University, Central Parkway, Newcastle upon Tyne, NE1 3BZ, UK; ^5^ Newcastle Eye Centre, Royal Victoria Infirmary, Newcastle upon Tyne, NE1 4LP, UK; ^6^ Wellcome Trust Centre for Mitochondrial Research, Institute of Neuroscience, Newcastle University, Newcastle upon Tyne, NE1 3BZ, UK; 7 ^7^Department of Experimental Psychology, University of Cambridge, CB2 3EB, UK

Sir,

We read with interest the article entitled ‘*OPA1*-related auditory neuropathy: site of lesion and outcome of cochlear implantation’ published by Santarelli and colleagues (2015). Hearing loss is a recognized symptom of a number of mitochondrial diseases and can result from neuronal (auditory nerve, brainstem, auditory cortex) or cochlear dysfunction. Although there have been isolated reports of auditory neuropathy in patients with mitochondrial disease (Ceranic and Luxon, 2004; Gamez and Minoves, 2006), Santarelli *et al.* (2015) were the first to show that it is an important cause of hearing impairment in a genetically defined subset of these patients.

Santarelli *et al.* (2015) comprehensively investigated the auditory phenotype in subjects with both missense (*n* = 10) and haploinsufficiency (*n* = 11) mutations in *OPA1*, which were first described in patients with autosomal dominant optic atrophy (Alexander *et al.*, 2000; Delettre *et al.*, 2000). Nine of eleven patients carrying haploinsufficiency variants had normal hearing whereas 9 of 10 patients carrying missense variants had a hearing impairment consistent with an auditory neuropathy. Cochlear implantation of six patients carrying missense *OPA1* mutations resulted in improved speech perception in all but one. Their findings indicate that the auditory neuropathy resulted from auditory nerve dysynchrony, and cochlear implantation subsequently improved auditory nerve synchrony and speech perception. This work provides a significant insight into the mechanism of hearing impairment and validates a treatment option in a cohort of patients with a classical form of mitochondrial disease.

Mutations in *OPA1* accounted for ∼3% of cases in a well-characterized patient cohort with multi-systemic mitochondrial disease (Gorman *et al.*, 2015). To determine whether the same mechanism is causing hearing impairment in other mitochondrial disorders, we studied four different genetically defined groups, including the most common mitochondrial DNA (mtDNA) mutation associated with deafness (m.3243A > G), and three nuclear genetic mitochondrial disorders caused by mutations in: *POLG*, *SPG7*, which is the most commonly diagnosed mutation in this patient group, and *OPA1* (Table 1). Our study had the relevant ethical and NHS approval. 

**Table 1:** Summary of patient demographics and clinical subtypes of the 30 patients enrolled in this study

**Disease group**	**Gene**	**Mean age, years (range) M:F**	**Variant**
MID *n* = 11	*MT-TL1*	55 (37–75); 2:9	mtDNA m.3243A>G
Polymerase gamma *n* = 4	*POLG*	61 (52–67); 7:3	Compound heterozygous p.Arg467Thr, p.Gly737Arg (*n* = 3) Compound heterozygous p.Trp748Ser, p.Gly737Arg (*n* = 1)
ARHSP *n* = 10	*SPG7*	54 (49–64); 0:4	Compound heterozygous inc. p.Ala510Val (*n* = 7) Heterozygous p.Gly349Ser (*n* = 1) Heterozygous p.Ala510Val (*n* = 2)
DOA *n* = 5	*OPA1*	56 (40–68); 3:2	Haploinsufficiency: c.2708_2711del (TTAG), p.V903fsX3 c.2294dupA p.Asn765fsX26 (*n* = 2) Missense c.869G>A p.Arg290His (*n* = 1)

ARHSP = autosomal recessive hereditary spastic paraplegia; DOA = autosomal dominant optic atrophy; MID = maternally inherited deafness and diabetes.

We undertook auditory phenotyping to interrogate both cochlear and neuronal hearing function. Pure tone audiometry at test frequencies of 0.25, 0.5, 1, 2, 4 and 8 kHz was used to ascertain overall subjective hearing level as a test of the whole auditory pathway. The pure-tone average, PTA, was defined as the average threshold across all of these test frequencies used. Neuronal function was tested with auditory brainstem responses (ABRs). Here, a click stimulus with alternating polarity was delivered at a suitable sensation level as to give a clear response. The sensation level was predetermined by the mean hearing level by ear at 2/4 kHz: ≤40 dB HL used click stimulus at 70 dB nHL, 40–60 dB HL used click stimulus at 80 dB nHL, >60 dB HL used click stimulus at 90 dB nHL. Contralateral masking was applied when required.

On average, 2000 responses were obtained on each side and these were bandpass filtered between 8 and 15 Hz. Waveforms and latencies of waves I–V were calculated and compared against age-matched values. Where the I–V interval was not available wave III–V and absolute V latency were calculated. Cochlear function was specifically tested using transient evoked otoacoustic emissions (TEOAEs) (click stimulus 84 dB SPL) for outer hair cell function and psychoacoustic tuning curves (PTCs) for both outer and inner hair cell function. Measurement of PTCs is the gold standard test for the detection of cochlear dead regions (Moore and Alcantara, 2001; Kluk and Moore, 2005). These regions of the cochlea, where the inner hair cells and/or auditory neurons are non-functional, are defined by the characteristic frequency of the auditory neurons adjacent to the affected region. A tone producing peak vibration in a dead region may be detected by ‘off frequency listening’, i.e. by spread of basilar-membrane vibration to a place in the cochlea where the inner hair cells/neurons are still functioning. The PTC is the level of a narrowband masker that is required to mask a sinusoidal signal measured as a function of the masker frequency. For normal-hearing individuals the tip of the PTC (the frequency at which the level of the masker is lowest) lies at or close to the signal frequency, whereas when the signal frequency falls in a dead region the PTC tip will be shifted from the signal frequency. The detection of cochlear dead regions may potentially elucidate hearing loss mechanisms. In conjunction with increased audiometric thresholds at defined frequencies a dead region may indicate specific impairment of inner hair cells. On the other hand, elevation of audiometric thresholds over a wide frequency range combined with broad PTCs without shifted tips is more likely to be a consequence of global metabolic cochlear disturbance resulting from dysfunction of the stria vascularis.

Using the ‘SWPTC’ programme, PTCs were measured using signal frequencies of 1, 2 and 4 kHz (Sek *et al.*, 2005; Sek and Moore, 2011). The four point moving average (4-PMA) method, in conjunction with visual inspection, was used to identify tip shifts away from the signal frequency. A tip shift of ±10% was taken as indicating a dead region at the signal frequency (tip frequency: signal frequency >1.10/<0.9), except when the PTC was very broad, giving a poorly defined tip.

## m.3243A > G patient group

Patients with the m.3243A > G mutation (*n* = 11) presented with a range of hearing levels: normal (*n* = 3/11); mild-to-moderate high-frequency loss (*n* = 5/11, mean PTA = 58 dB HL, age-corrected PTA = 39 dB HL); and severe-to-profound loss at all measured frequencies (*n* = 3/11, mean PTA = 91 dB HL, age-corrected PTA = 61 dB HL). Patients with pathological mtDNA mutations often have a mixture of mutated and wild-type mtDNA molecules (heteroplasmy). Although there was a positive correlation between mean age-corrected PTA and urinary heteroplasmy levels (range 25–66%), this did not reach statistical significance (r = 0.37, *n* = 10, *P* = 0.3). No relationship was found between hearing levels and blood heteroplasmy levels (range 3–30%).

**Figure 1:**
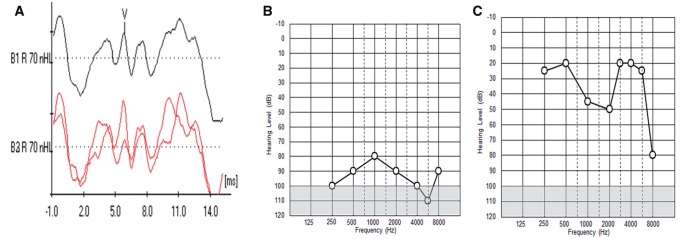
**Auditory brain stem responses and pure tone audiograms.** (**A**) Auditory brainstem response (ABR) from the right ear of a representative m.3243A > G patient showing normal waveform morphology and latencies (wave V marked). The black line represents an average of individual readings (red lines). (**B**) Pure tone audiogram showing profound hearing loss at all tested frequencies (representative of hearing in three patients with m.3243A > G). (**C**) Pure tone audiogram showing predominantly mid-frequency loss [polymerase gamma (*POLG*) variant p.Arg467Thr, p.Gly737Arg].

TEOAEs were absent for all patients, except for those with normal hearing. ABRs were not recordable for the two patients with profound hearing loss. The mean interpeak latencies of ABR waves I-III (2.4 ms, range 2.1–2.6), III-V (1.74 ms, range 1.5–1.9) and I-V (4.0 ms, range 3.9–4.4) were within the normal range for the remaining patients (normal ranges 1.5–2.6 ms, 1.3–2.7 ms, 3.3–4.7 ms, respectively) (Chalak *et al.*, 2013) (Fig. 1A and B). 

**Figure 2:**
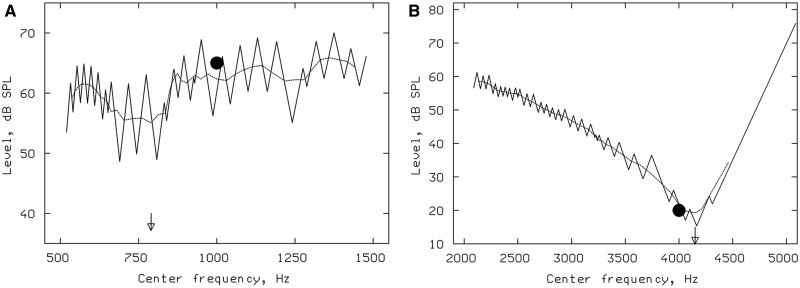
**Psychophysical analysis.** (**A**) Psychophysical tuning curve (PTC) determined using ‘SWPTC’ program showing a significant tip shift below the signal frequency. Solid line represents raw PTC, dashed line represents the 4-PMA, filled circle indicates the signal frequency (1 kHz) and the arrow indicates the frequency of the tip of the PTC, estimated from the 4-PMA (790 Hz). (**B**) PTC analysis in a representative case of autosomal recessive hereditary spastic paraplegia (ARSHP) showing PTC tip analysed by 4-PMA. The tip of the PTC falls close to the signal frequency (4 kHz, green circle).

PTC analysis was performed for 9/11 patients. Two patients were unable to complete the test due to the severity of their hearing impairment. For patients with normal or mild hearing loss, no tip shifts were detected (mean tip ratio 1.06, range 1.04–1.14; the tip shift of 1.14 was associated with a very broad PTC with an ill-defined tip). For patients with moderate-to-severe hearing loss, two patients showed tip shifts at 1 kHz consistent with dead regions (tip shift ratios 0.79 and 1.11) (Fig. 2A). 

## *POLG* patient group

Hearing loss was present for all *POLG* patients studied (*n* = 4/4, mean PTA = 39 dB HL, age corrected PTA 14 dB HL). Moderate high-frequency hearing loss was present in two patients and one patient had a moderate loss at all measured frequencies. One patient showed a predominantly moderate mid-frequency loss (Fig. 1C). TEOAEs were absent for all patients. ABR latencies were within the normal range for three patients (mean interpeak latencies I-III 2.1 ms, range 1.6–2.6 ms, III-V 1.9 ms, range 1.4–2.4 ms, I-V 4.2 ms, range 4–4.4 ms). One patient with moderate high-frequency loss had delayed wave I-III interpeak latencies of 3.15 ms and wave I-V latencies of 5.25 ms, but with absent TEOAEs suggestive of primary cochlear dysfunction. PTC analysis was performed for four patients and revealed no dead regions (mean tip ratio 1.06, range 1–1.09).

## *SPG7* patient group

The SPG7 patients (*n* = 10) either had normal hearing (*n* = 5/10) or a moderate high-frequency hearing loss (*n* = 5/10, mean PTA = 30 dB HL, age corrected PTA 8 dB HL). TEOAEs were present for 9/10 patients. The interpeak latencies of ABR waves I-III (mean latency 2.2 ms, range 1.8–2.4 ms), III-V (mean 1.84 ms, range 1.6–2.2 ms) and I-V (mean 4.0 ms, range 3.7–4.5 ms) were within the normal range for all patients. PTC analysis was performed for 9/10 patients. One patient was unable to perform the test due to ataxia. No dead regions were detected (mean tip ratio 1.02, range 0.98–1.12; the tip shift of 1.12 was associated with a broad PTC). See Fig. 2B for a representative PTC.

## *OPA1* patient group

We also included a cohort of patients with *OPA1* mutations harbouring haploinsufficiency (*n* = 4) or missense (*n* = 1) *OPA1* mutations. Patients carrying haploinsufficiency mutations had either normal hearing (*n* = 3) or a mild high-frequency loss (*n* = 2, mean PTA = 31 dB HL, age corrected PTA 5 dB HL). TEOAEs were absent for one patient with high-frequency loss. One patient with a missense *OPA1* mutation had a moderate high-frequency loss (mean PTA = 51 dB HL, age corrected PTA = 21 dB HL) with absent TEOAEs. The interpeak latencies of ABR waves I-III (2.1 ms, range 1.9–2.2 ms), III-V (1.7 ms, range 1.3–1.8 ms) and I-V (3.6 ms, range 3.4–3.9) were in the normal range for all patients. PTC analysis was performed for 5/5 patients and did not reveal any dead regions (mean tip ratio 1.03, range 0.98–1.1).

## Discussion

In agreement with previous studies, we found hearing levels for patients carrying the m.3243A > G mutation in the range from normal to profound loss, with absent TEOAEs but preserved ABRs, indicating that the hearing loss is cochlear in origin (Chinnery *et al.*, 2000). However, heteroplasmy levels of the m.3243A > G mutation did not account for a significant proportion of the phenotypic variation (Chinnery *et al.*, 2000; Uimonen *et al.*, 2001), perhaps because of the limited size of this study group. For the first time, we show that hearing loss is also a common feature in *POLG* disease and similar to m.3243A > G, this seems to be cochlear in origin. Likewise, mild cochlear hearing impairment or normal hearing was found in our *SPG7* and *OPA1* patient cohort.

In conclusion, our data suggest that both mtDNA mutations and a range of nuclear-genetic mitochondrial disorders cause hearing loss through cochlear dysfunction. The presence of dead regions in a small number of patients carrying the m.3243A > G mutation suggests that mtDNA point mutations cause specific dysfunction of inner hair cells. On the other hand, the absence of cochlear dead regions with loss of TEOAEs, but with normal ABRs, suggest that the hearing loss in patients with *POLG*, *SPG7* and *OPA1* mutations is more likely due to global cochlear dysfunction. Although we cannot exclude the possibility that severely impaired patients have auditory nerve dysfunction (masked by the severe cochlear defect), we found no evidence of isolated auditory nerve impairment in our patient cohort.

Hearing loss has a negative impact on quality of life and it is often overlooked in patients with mitochondrial disease who can present with a range of complex heterogeneous symptoms. Our data confirm that sensorineural hearing loss is an important pathological feature in a range of mitochondrial diseases, including patients with *SPG7* and *POLG* mutations, who have not been systematically studied before. Further improvements in understanding the pathogenesis and natural history of these disorders are important to provide better prognostic information to patients and to inform future trial design.

## Funding

P.F.C. is a Wellcome Trust Senior Fellow in Clinical Science (101876/Z/13/Z), and a UK NIHR Senior Investigator, who receives support from the Medical Research Council Mitochondrial Biology Unit (MC_UP_1501/2), the Wellcome Trust Centre for Mitochondrial Research (096919Z/11/Z), the Medical Research Council (UK) Centre for Translational Muscle Disease research (G0601943), EU FP7 TIRCON, and the National Institute for Health Research (NIHR) Biomedical Research Centre based at Cambridge University Hospitals NHS Foundation Trust and the University of Cambridge. P.J.K. is a Wellcome Trust Clinical research fellow (101700/Z/13/Z). P.Y.W.M. is supported by a Clinician Scientist Fellowship Award (G1002570) from the Medical Research Council (UK), and also receives funding from Fight for Sight (UK) and the UK National Institute of Health Research (NIHR) as part of the Rare Diseases Translational Research Collaboration. The views expressed are those of the author(s) and not necessarily those of the NHS, the NIHR or the Department of Health. We acknowledge Medical Research Council (MRC) Centre Mitochondrial Disease Patient Cohort: A Natural History Study and Patient Registry (REC ref number:13/NE/0326) for patient screening and data provision. We are grateful to Alex Bright, Research Nurse, for her work recruiting individuals with the m.3243A>G and *POLG* mutations.
